# Physician distribution across China’s cities: regional variations

**DOI:** 10.1186/s12939-021-01503-5

**Published:** 2021-07-13

**Authors:** Xuexin Yu, Wei Zhang, Jersey Liang

**Affiliations:** 1 West China Biomedical Big Data Center, West China Hospital, Sichuan University, 37 Guoxue Alley, Chengdu, 610040 Sichuan China; 2 Department of Health Management and Policy, School of Public Health, 1415 Washington Heights, Ann Arbor, MI 48109-2029 USA

**Keywords:** Physician supply, Health equity, Regional variations, Urban China

## Abstract

**Background:**

Distribution of physicians is a key component of access to health care. Although there is extensive research on urban-rural disparities in physician distribution, limited attention has been directed to the heterogeneity across urban areas. This research depicts variations in physician density across over 600 cities in the context of China’s rapid urbanization.

**Methods:**

Data came from National Census Surveys and China statistical yearbooks, 2000–2003, and 2010–2013. Cities were characterized in terms of not only administrative level but also geographic regions and urban agglomerations. We analyzed variations in physician supply by applying generalized estimating equations with an ordinal logistic linking function.

**Results:**

Although overall physician density increased between 2003 and 2013, with population and socioeconomic attributes adjusted, physician density declined in urban China. On average, urban districts had a higher physician density than county-level cities, but there were regional variations. Cities in urban agglomerations and those outsides did not differ in physician density.

**Conclusion:**

Despite the reduced inequality between 2003 and 2013, the growth in physician density did not appear to be commensurate with the changes in population health demand. Assessment in physician distribution needs to take into account heterogeneity in population and socioeconomic characteristics.

**Supplementary Information:**

The online version contains supplementary material available at 10.1186/s12939-021-01503-5.

## Introduction

Equitable distribution of physicians is a key component of access to health care [1, 2]. Prior studies from high-income countries have focused on the association between physician density and a community’s socioeconomic and demographic characteristics, including residents’ average household income [3, 4], education [5], population density [6, 7], racial composition [6], and urban versus rural settings [4, 8, 9]. Earlier studies in China have also provided some evidence on geographic inequality in physician distribution [10–14]. For example, Song et al. [11] found the Gini coefficient of pediatricians distribution in Eastern China (0.25) was higher than those in Western (0.14) and Central China (0.12).

Despite a fair number of descriptive studies in China, the vast majority of analytic studies on physician distribution were based on data derived from developed nations. Whether their findings could be generalized to developing countries, such as China, is unknown, since the financing, healthcare delivery, and social contexts differ substantially between developing and developed nations. Further, previous research has focused on the disparities in physician distribution between urban and rural areas, with limited attention to the heterogeneity across cities [15]. This is an important gap, particularly in China, as Anand et al. [14] noted that, in urban areas (Gini coefficient: 0.21), where more than half of Chinese resided, the inequality in the distributions in physicians was twice as high as that in the rural areas (Gini coefficients: 0.09). Recent evidence has also suggested that physician density in urban China increased from 2.5 per 1000 populations in 2005 to 3.8 in 2015, whereas physician density in rural areas was consistently under 2 [16].

### Health care reforms in China

China’s health care system has gone through several transformations since the early 1950s. Until the early 1980s, the government-owned and operated all health care facilities and health services were essentially free. In 1984, China shifted public to private financing for health care, which left the vast majority of the population uninsured and increased health expenditures. To mitigate popular discontent, in 2003, the Chinese government introduced health insurance for rural and urban residents, following the reform of health insurance for urban employees in the mid-1990s [17].

Since 2009, China has implemented several initiatives to curb the escalation of health expenditures, improve access to health care, and decrease health inequality [18]. They include universal health insurance coverage [18, 19], essential medicine policies [20], providing the basic public health service package [21], reforming public hospitals [22], and strengthening the capacity of primary care [23]. China has managed to extend a basic health care safety net for more than 95% of its population in 2012, despite being a developing country with approximately 1.4 billion people [17, 24]. Although the benefits package of the health insurance differed among urban and rural residents, the integration of urban and rural basic health care systems, along with several financial protection policies for vulnerable populations, is underway to bridge the gap [19].

Nonetheless, stark health disparities remain, for which the distribution of health care human resources is a major cause [14, 18]. China has greatly expanded the enrollment of medical students since 1988, and adopted various medical education programs, typically ranging from (a) 3-year training in a vocational school or a junior college to (b) a 5-year program in a university followed by a 3-year hospital-based residency [16, 25, 26]. Students in 5-year programs increased from 0.32 million to 3.05 million between 1999 and 2018, whereas students in 3-year programs increased from 0.53 million to 1.21 million during the same period [27]. According to a recent projection of the distribution of physicians by education level between 2015 and 2035 [16], the share of physicians with a bachelor’s degree or above would increase from 47% in 2015 to 54% in 2035. Nonetheless, a significant proportion of medical graduates do not practice medicine after graduation [14]. A lack of effective incentives to attract and retain human health resources, particularly in underserved and poor areas, remains a major challenge [23].

### Urbanization in China

Since the market reform in 1978, an estimated 262 million people have migrated from rural areas to urban areas because of greater job opportunities, better social welfare, and improved living conditions [28, 29]. Cities in China can be divided into three categories: provincial-level cities, prefecture-level cities, and county-level cities, reflecting the substantial heterogeneity in economic development, population density, and social welfare [30]. Also, they are hierarchical because provincial/prefecture-level cities encapsulate densely populated urban districts as well as sparsely-populated county-level cities and counties. Urban districts have been traditionally recognized as the narrowly-defined provincial/prefectural-level cities since social welfare in urban districts is better than county-level cities and counties [30, 31].

Furthermore, there exist significant economic disparities across regions in China. Regions close to coastlines, where the market-oriented reform was first initiated, such as the Eastern Region, are economically more developed relative to other regions [32]. Thus, Eastern China is recognized as more attractive to physicians in comparison with Western China, which has experienced a significant brain drain [13].

As a vehicle of its economic development strategy, China has established a number of urban agglomerations (U.A.s). A U.A. or city cluster consists of several metropolis or large cities, which form a multi-layer group centered on one or two most prosperous cities [33]. Representing a more advanced form of urbanization, each U.A. has its dominant industries, connected transportation networks, and similar social public welfare [34]. Geographic information of regions and U.A.s is presented in Supplemental Materials Fig. 1.

## Objectives and hypotheses

This research aims to analyze urban disparities in physician density in China. As a guide for data analysis and interpretation of findings, we offered four hypotheses.

First, equality in physician density across cities in China improved significantly between 2003 and 2013 (H_1_), given the significant growth of human healthcare resources and substantial economic growth [14, 28].

Second, physician density is higher in urban districts than in county-level cities, even when demographic and socioeconomic characteristics are controlled (H_2_). Disparities in physician distribution across cities could be driven by the uneven distribution of supply factors that affect physicians’ location choices, including opportunities for education and professional prestige [15]. These factors may not be fully adjusted by including socioeconomic and population attributes.

Third, the urban disparities in physician distribution may exacerbate across regions (H_3_) as there are substantial regional differences in socioeconomic development because of structural and long-term factors including education, regional labor supply, and geographic location [35]. For instance, the national government adopted preferential policies such as the Open and Reform, benefiting the coastal area disproportionally, and the Western Development policy, creating favorable conditions for investment in Western provinces [32].

Fourth, recent initiatives of creating urban agglomerations across China to promote economic growth are associated with increased physician density (H_4_). There is some evidence of the geographic spillover effect of urban agglomerations as an increasing function of the overall human capital level, foreign direct investment share, and its “synergy” with nearby incumbent firms [36].

## Methods

### Data source

Data came from China City Statistical Yearbooks and National Census Surveys, 2000–2003, and 2010–2013. We obtained the total numbers of physicians in 2003 and 2013, and the land area and GDP in 2000 and 2010 from the China City Statistical Yearbooks. We derived population attributes (e.g., total population, female-to-male ratio, migrant population) in the years 2000 and 2010 from the National Census Surveys.

### Measures

To measure physician density, we calculated the number of physicians per 1000 population [37]. China City Statistical Yearbooks identified physician supply according to the number of physicians affiliated with local medical institutions. In our calculation, we included physicians and assistant physicians who passed the National Physician or Assistant Physician License Examination, regardless of their educational attainment, although, in 2011, about one-half of the 2.02 million physicians (48.7%) and 440 thousand assistant physicians (52.8%) did not have a bachelor’s degree [38]. Physicians included in our analyses work at different-level medical institutions, e.g., general hospitals (45%), specialist hospitals (9%), primary care centers (28%), and nursing care centers (0.2%) [27]. These physicians include those practicing western medicine and traditional Chinese medicine (16% of physicians and 14% of assistant physicians) [27]. In urban China, assistant physicians practice under the supervision of physicians and cannot prescribe medication while they may practice independently in rural areas [39].

Data on population reflected the actual number of residents in each city, including the migrant population but excluding those registered in the Hukou system while seeking jobs and living in other areas. Physician density was highly skewed to the left, i.e., dominated by cities with lower physician density (Supplemental Materials Fig. 2). To characterize its distribution better, we divided it into three groups: low-density (bottom 1/3; 0–1.48 physicians per 1000 population), medium-density (middle 1/3, 1.49–2.46 physicians per 1000 population), and high-density (top 1/3; 2.47–9.68 physicians per 1000 population).

Due to data limitations, we merged urban districts in a given provincial-level or prefecture-level city and treated them as one study unit, whereas each county-level city was treated as a single study unit. The geographic region of each city was categorized as (a) Eastern China, (b) Northeastern China, (c) Central China, and (d) Western China [40]. We identified cities in four major U.A.s, including (a) Beijing-Tianjin-Hebei U.A. consisting of 13 cities with Beijing, the capital city of China, as the center; (b) the Yangtze River Delta U.A. including 26 cities with China’s most developed city, Shanghai, in the center; (c) the Pearl River Delta U.A. with nine cities that are centered around Shenzhen and Guangzhou; and (d) the Chengdu-Chongqing U.A. consisting of 16 cities with two large cities in Western China, Chengdu and Chongqing, as central cities [41]. We combined four U.A.s in one category since it was not feasible to differentiate them because of the small number of cities included in each U.A.

Socioeconomic characteristics included GDP per capita and the proportion of the population with a high school education or above. Demographic attributes including population density (i.e., population per land area), female-to-male ratio (the number of female individuals/the number of male individuals, %), proportion of the migrant population, proportion of minority population, proportion of persons aged under 15, and proportion of persons aged over 65, were introduced as covariates to reflect population health demand [4, 42, 43].

Overall, 16 cities in 2000–2003 and 21 cities in 2010–2013 were excluded because of missing data. Two observations were available for 614 cities, whereas 50 cities had only one observation.

### Data analyses

To gauge the inequality in physician distribution, we first calculated Gini coefficients for 2003 and 2013 [44]. This was supplemented by descriptive analyses of physician density in conjunction with the demographic and socioeconomic characteristics in 2003 and 2013. To evaluate differences in physician distribution over time, we applied generalized estimating eqs. (G.E.E.) with an ordinal link function, which is more appropriate for a dependent variable with a skewed distribution [45]. Population and socioeconomic attributes were introduced as covariates. Variance inflation factors ranged from 1.13 to 3.72, indicating moderate but acceptable multicollinearity.

To evaluate the robustness of our results, several supplemental analyses were performed. First, as county-level cities could be promoted to prefectural-level cities between 2003 and 2013, we excluded such cities and reanalyzed variations in physician density. Second, because county-level cities and urban districts were nested within the given provincial/prefectural-level city, multilevel ordinal logistic analyses were performed to account for repeated observations of each study area and the clustering effects among county-level cities/urban districts within the same provincial/prefectural-level city. Last, the four provincial-level municipalities or top-tier cities (i.e., Beijing, Tianjin, Shanghai, and Chongqing) may differ significantly from other prefecture-level cities in their socioeconomic development. Hence, we reanalyzed the data by excluding these four municipalities. Results from these supplemental analyses were similar (Supplemental Materials Tables 3–5). All analyses were performed with SAS University Edition (SAS Institute, Inc., Cary, NC, USA).

## Results

### Descriptive analyses

Overall, physician distribution across cities improved between 2003 and 2013 (H_1_). Specifically, the decline in Gini coefficients between 2003 and 2013 from 0.31 to .025 suggested that inequality in physician distribution in China’s cities improved during this period (Supplemental Materials Fig. 3).

According to the descriptive analysis, physician density in China’s cities increased from 1.80 in 2003 to 2.48 in 2013. County-level cities experienced a 41% increase in physician density (from 1.36 to 1.92), whereas urban districts had a 30% increase (from 2.47 to 3.20) (Table 1). Furthermore, there appeared to be some regional variations across urban districts and county-level cities. For instance, urban districts in Western China had a lower physician density (2.17 in 2003 and 2.91 in 2013) than other regions. In contrast, county-level cities in Western China had the highest physician density (1.63 in 2003 and 2.36 in 2013) of their kinds among all regions. Finally, physician density varied substantially across U.A.s (Table 2). Specifically, Beijing-Tianjin-Hebei U.A., Yangtze River Delta U.A., and Pearl River Delta U.A. had a higher physician density than Chengdu-Chongqing U.A. and cities which were not part of any U.A.

**Table 1:** Physician supply and socioeconomic characteristics of study area (mean values), 2003–2013

		2003		2013	
Region	Variable	Urban district	County-level city	Urban district	County-level city
Overall		*N* = 272	*N* = 368	*N* = 281	*N* = 357
	Physician density	2.47	1.36	3.20	1.92
	High school education or above (%)	21.13	11.50	34.12	21.51
	GDP per capita (10,000 CNY)	1.46	0.95	3.76	3.43
	Migrant (%)	21.25	10.43	27.97	16.38
Central		*N* = 79	*N* = 88	*N* = 80	*N* = 84
	Physician density	2.62	1.33	3.57	1.85
	High school education or above (%)	21.97	10.84	36.92	22.10
	GDP per capita (10,000 CNY)	1.14	0.67	3.26	3.25
	Migrant (%)	19.29	6.66	24.7	10.76
Northeastern		*N* = 34	*N* = 56	*N* = 34	*N* = 55
	Physician density	2.73	1.61	2.87	1.68
	High school education or above (%)	24.77	13.09	36.88	19.67
	GDP per capita (10,000 CNY)	1.38	0.81	4.08	3.29
	Migrant (%)	19.74	9.43	24.00	12.79
Western		*N* = 72	*N *= 80	*N *= 84	*N* = 80
	Physician density	2.17	1.63	2.91	2.36
	High school education or above (%)	17.63	12.84	29.45	23.78
	GDP per capita (10,000 CNY)	1.08	0.90	3.21	3.31
	Migrant (%)	17.20	15.15	27.27	21.51
Eastern		*N* = 87	*N* = 144	*N* = 83	*N* = 138
	Physician density	2.48	1.13	3.28	1.81
	High school education or above (%)	21.84	10.53	35.01	20.57
	GDP per capita (10,000 CNY)	2.11	1.20	4.59	3.67
	Migrant (%)	26.97	10.51	33.46	18.24

Note: All demographic attributes are available from Supplemental Material Table 1. GDP was adjusted by Consumer Price Index

**Table 2:** Physician supply and socioeconomic characteristics of urban agglomerations (mean values), 2003–2013

U.A.	Characteristic	2003	2013
Beijing-Tianjin-Hebei U.A.		*N* = 11	*N* = 13
	Physician density	3.30	4.15
	High school education or above (%)	26.08	45.68
	GDP per capita (10,000 CNY)	1.61	4.62
	Migrant (%)	23.45	32.18
Yangtze River Delta U.A.		*N* = 26	*N* = 24
	Physician density	2.54	3.04
	High school education or above (%)	23.50	33.55
	GDP per capita (10,000 CNY)	2.97	5.37
	Migrant (%)	26.25	33.62
Pearl River Delta U.A.		*N* = 8	*N* = 8
	Physician density	2.45	2.64
	High school education or above (%)	24.43	38.05
	GDP per capita (10,000 CNY)	2.44	5.99
	Migrant (%)	55.73	55.51
Chengdu-Chongqing U.A.		*N* = 14	*N* = 15
	Physician density	1.68	2.44
	High school education or above (%)	12.06	25.45
	GDP per capita (10,000 CNY)	0.69	2.77
	Migrant (%)	11.93	20.45
Areas not in any U.A.		*N* = 581	*N* = 578
	Physician density	1.77	2.42
	High school education or above (%)	15.00	26.27
	GDP per capita (10,000 CNY)	1.07	3.47
	Migrant (%)	13.88	20.29

Note: U. A. represents urban agglomeration. All demographic attributes are available from Supplemental Material Table 2. GDP was adjusted by Consumer Price Index

### Multivariate analyses

Table 3 presents the results from the G.E.E. regression analysis of physician density in 2003 and 2013. In sharp contrast with the results from our descriptive analysis, odds of having a higher physician density were significantly greater in 2003 than in 2013 (OR = 0.17, 95% CI = 0.07–0.38), suggesting a possible deterioration in access over time, when heterogeneity in population and socioeconomic characteristics were adjusted (Model 1in Table 3).

**Table 3:** GEE regression analysis of physician density, 2003–2013

Characteristic	Model 1 OR (95% CI)			Model 2 OR (95% CI)
Intercept2	0.01 (0.01 to 0.03) ^***^			0.02 (0.01 to 0.04) ^***^
Intercept1	0.17 (0.07 to 0.38) ^***^			0.21 (0.09 to 0.48) ^***^
Year 2013 (vs. year 2003)	0.49 (0.34 to 0.72) ^***^			0.50 (0.33 to 0.75) ^***^
Urban district (vs. county-level city)	2.62 (1.84 to 3.73) ^***^			
Geographic location				
Eastern (vs. Western)	0.52 (0.36 to 0.77) ^**^			
Northeastern (vs. Western)	0.73 (0.45 to 1.21)			
Central (vs. Western)	0.65 (0.44 to 0.95) ^*^			
Geographic location & Administrative level				
County-level city x Western				Ref.
Urban district x Eastern				1.49 (0.81 to 2.74)
County-level city x Eastern				0.32 (0.20 to 0.52) ^***^
Urban district x Northeastern				1.09 (0.49 to 2.38)
County-level city x Northeastern				0.62 (0.33 to1.15)
Urban district x Central				1.47 (0.80 to 2.71)
County-level city x Central				0.42 (0.25 to 0.70) ^***^
Urban district x Western				1.53 (0.93 to2.52)
U.A. (vs. non-U.A.)	0.87 (0.51 to 1.49)			0.75 (0.44 to 1.29)
High school education or above (%)	1.21 (1.17 to 1.25) ^***^			1.21 (1.17 to 1.25) ^***^
GDP per capita (10,000 CNY)	1.15 (0.83 to 1.59)			1.02 (0.96 to 1.08)
Population density (/1000 km^2^)	0.99 (0.78 to 1.25)			0.96 (0.76 to 1.22)
Female-to-male ratio (in a unit of 10%)	0.84 (0.70 to 0.99) ^*^			0.86 (0.73 to 1.01)
Migrant (in a unit of 10%)	1.08 (0.91 to 1.28)			1.07 (0.90 to 1.26)
Minority (in a unit of 10%)	1.02 (0.93 to 1.12)			0.99 (0.91 to 1.09)
Aged under 15 (in a unit of 10%)	0.93 (0.81 to 1.07)			0.94 (0.83 to 1.07)
Aged over 65 (in a unit of 10%)	1.57 (0.66 to 3.72)			1.78 (0.70 to 4.54)

Note: ^***^ *P* < .001; ^**^ *P* < .01; ^*^ *P* < .05. U.A. represents urban agglomeration. County-level city x Western represents county-level cities in Western China. GDP was adjusted by Consumer Price Index

Per our hypothesis, urban districts were more likely to have a higher physician density (OR = 2.62, 95% CI = 1.84–3.73) relative to county-level cities. This association remained robust even with population and socioeconomic attributes controlled (H_2_).

Regions mattered but in an unexpected direction (Model 1). Specifically, in comparison with Western China, economically more developed Eastern China (OR = 0.52, 95% CI = 0.36–0.77) and Central China (OR = 0.65, 95% CI = 0.44–0.95) were significantly less likely to have a higher physician density. There were no significant differences in physician density between Northeastern China and Western China. These findings provide some evidence that the difference between urban districts and county-level cities in physician density varied across regions, as we hypothesized (H_3_).

According to results pertaining to Model 2, regional differences existed among county-level cities but not urban districts. Relative to county-level cities in the less developed Western China, county-level cities in Eastern China had lower odds of being in a higher physician-density group (OR = 0.32, 95% CI = 0.20–0.52). Likewise, county-level cities in Central China (OR = 0.42, 95% CI = 0.25–0.70) were at a substantial disadvantage in comparison with their counterparts in Western China.

Finally, contrary to our hypothesis (H_4_), urban districts in the four urban agglomerations did not differ from areas not included in the U.A.s (Model 2 in Table 3). Because the Chengdu-Chongqing U.A. had a lower physician density and was less developed socioeconomically relative to the other three U.A.s, it appeared to be more like cities outside any U.A. (Table 2). Hence, we conducted an additional analysis by excluding the Chengdu-Chongqing U.A.from the U.A.s. The results were similar.

### Predicted probabilities

The magnitude of the odds ratio is difficult to interpret because of the arbitrary scaling factor [46]. Consequently, based on the models in Table 3, we derived the predicted probabilities for high, medium, and low physician density by urban districts/county-level cities, regions, and urban agglomeration (Table 4), while controlling for socioeconomic and demographic covariates by constraining them at their mean values.

**Table 4:** Predicted probabilities for physician density after controlling for population and socioeconomic covariates, 2003–2013

Region	2003			2013		
	Low density	Medium density	High density	Low density	Medium density	High density
	[0.00–1.48]	[1.49–2.46]	[2.47–9.68]	[0.00–1.48]	[1.49–2.46]	[2.47–9.68]
Model 1						
Urban district	0.17	0.55	0.28	0.29	0.55	0.16
County-level city	0.34	0.53	0.13	0.52	0.42	0.07
Eastern China	0.17	0.55	0.28	0.29	0.55	0.16
Northeastern China	0.13	0.52	0.35	0.23	0.56	0.21
Central China	0.14	0.54	0.32	0.25	0.56	0.19
Western China	0.09	0.48	0.43	0.18	0.56	0.27
Non-U.A.	0.29	0.43	0.28	0.29	0.55	0.16
U.A.	0.32	0.43	0.25	0.32	0.54	0.14
Model 2						
Urban district x Eastern	0.14	0.54	0.33	0.24	0.57	0.19
County-level city x Eastern	0.42	0.49	0.09	0.59	0.36	0.05
Urban district x Northeastern	0.18	0.56	0.26	0.30	0.55	0.15
County-level city x Northeastern	0.27	0.56	0.17	0.43	0.48	0.09
Urban district x Central	0.14	0.54	0.32	0.24	0.57	0.19
County-level city x Central	0.36	0.52	0.12	0.53	0.41	0.06
Urban district x Western	0.13	0.54	0.33	0.23	0.57	0.20
County-level city x Western	0.19	0.57	0.25	0.32	0.54	0.14

Note: Low, medium, and high density respectively represent physician density ranging from 0.00 to 1.48, 1.49 to 2.46, and 2.47 to 9.68 per 1000 population. Probabilities were derived on the basis of the models in Table 3 with covariates conditioned at the following values: High school education or above = 21.32%, GDP per capita = 2.89, population density = 779.80 (/km^2^), female-to-male ratio = 96.81%, migrant = 18.25%, minority = 7.88%, aged under 15 = 18.77%, aged over 65 = 7.94%. UA represents urban agglomeration. County-level city x Western represents county-level cities in Western China

First, adjusting for population and socioeconomic attributes, the decline in physician density between 2003 and 2013 is further illustrated by the estimated probabilities (Table 4). For both urban districts and county-level cities, probabilities for low physician density increased from 2003 to 2013. In contrast, probabilities for high physician density declined substantially during the same period.

Regarding the differences between urban districts and county-level cities, urban districts had a greater probability of high physician density (0.28 in 2003 and 0.16 in 2013) relative to county-level cities (0.13 in 2003 and 0.07 in 2013). Western China had a much higher probability for high physician density (0.44 in 2003 and 0.27 in 2013) relative to other regions, which had probabilities for high physician density ranging respectively from 0.28 to 0.35 in 2003 and from 0.16 to 0.21 in 2013 (Table 4).

The disparities among county-level cities across regions are also exhibited by the predicted probabilities. As shown in Table 4, probabilities in high, medium, and low physician density groups are quite similar among urban districts across the four regions. In contrast, county-level cities in East China and Central China had the lowest probabilities for high physician density (0.09 and 0.12 in 2003; 0.05 and 0.06 in 2013) among all county-level cities.

Last, cities in and those outside an urban agglomeration exhibited similar probabilities in terms of low, medium, and high physician density, although probabilities for a high physician density were substantially diminished.

## Discussion

By analyzing data from over 600 cities, the present study provides an in-depth depiction of physician distribution in China. To the best of our knowledge, this is the first examination of physician distribution across China’s cities in conjunction with regional differences and urban agglomeration while considering the heterogeneity in population and socioeconomic characteristics over time.

Based on descriptive analyses, physician density increased across urban China. This conclusion was, however, not supported by multivariate analyses. Further analysis revealed that, in predicting physician density, the regression coefficient associated with the binary measure of the year 2013 flipped from positive to negative when the proportion of the population with high school education entered in the model as a covariate. This could be a result of the high correlations among these three variables. Specifically, the proportion of high school graduates was highly correlated with not only physician density (r = .683, *P* < .0001) but also the binary measure of the year 2013 (r = .526, *P* < .0001). The substantial improvement in the level of education between 2003 and 2013 may lead to a significant increase in the demand for physicians, supported by studies suggesting that higher education attainment was associated with increased health service utilization [47–49]. However, the increase in physician density might have lagged behind the improvement of educational attainment across China’s cities. Consequently, physician density declined when one adjusted educational attainment of the urban populations. This is true even though overall physician density increased between 2003 and 2013.

Prior research on the disparities in physician distribution has directed limited attention to physician density among cities. The present study contributes to our understanding of how physician density varies between urban districts and county-level cities in China by showing that urban districts with significantly higher physician density than county-level cities, even with population and socioeconomic characteristics taken into account. Above and beyond the population and socioeconomic attributes included in our models, compared with county-level cities, urban districts may be more attractive to physicians because of community amenity, greater employment and income potential, opportunities for professional development, and better working conditions [15, 50, 51].

The present research provides further insights regarding inequality in physician density across China’s cities by highlighting their regional variations. First, regional differences existed only among county-level cities but not urban districts. Second, Eastern and Central China regions had the lowest probabilities of high physician density relative to other regions. As county-level cities in Central China had lower education and GDP per capita (Table 1), it was not surprising that they were less likely to have high physician density. More surprising was that county-level cities in Eastern China, whose county-level cities had the highest GDP per capita across regions, were disadvantaged in terms of high physician density.

We offered the following hypotheses to account for the lower physician density in county-level cities in Eastern China. First, urban districts in Eastern China may attract physicians from neighboring county-level cities because of the advanced transportation systems, which reduce the time cost of migration of physicians [38]. This possibility was supported by an observed positive correlation between the length of highway and health professionals’ distribution [52]. Additionally, numerous tertiary hospitals in urban districts in Eastern China may lead to a “Matthew effect” that attracts an increasing number of physicians from nearby communities. Since career prospect plays an important role for physicians in choosing a workplace [6, 8, 53], physicians may benefit from urban districts in Eastern China, which had 33.52% of China’s tertiary hospitals (1399 in total) in 2011, while Central, Northeastern, and Western regions only took up 28.59, 13.22, and 24.73%, respectively [54].

The present research offered a rare opportunity to assess the linkages between physician density and U.A.s in China. According to our findings, U.A. explains little variation in physician density beyond what had been accounted for by the population and socioeconomic measures included in our models. Nonetheless, our binary measure of the U.A.s was a crude measure that masks the heterogeneity among the four U.A.S. Because of the limited number of cities within each U.A., we were unable to estimate the differences across the four agglomerations versus other cities that were not part of any agglomeration. Further, China has more than 20 U.A.s [55]. The definition of U.A.s varies, and there is no single national standard measure of U.A.s to date [55]. Hence, we conducted additional analyses by creating an alternative measure of U.A.s, which included several other urban agglomerations that consisted of metropolitan cities like Wuhan, Zhengzhou, Xi’an, and Qingdao. Results, however, remained consistent with our main findings.

Two major policy implications could be drawn from our research. First, assessment of equality in physician distribution needs to take into account not only the heterogeneity in population and socioeconomic characteristics but also additional dimensions such as regional variations and urban agglomeration. For most developing countries where growing population health demand exists, the increase in health care resources should be evaluated in the context of social development. The strategy is also essential for developed countries that experience rapid population aging with increasing demand for older care. Second, county-level cities in Central and East China may require extra support in recruiting and retaining physicians. Policy responses to address the uneven distribution of physicians include selection, education, regulation, incentives, and reforms of healthcare delivery [15]. Well-defined recruitment and education strategies, e.g., targeting students with a rural background, hold great promise.

This study has several limitations. First, because of the lack of data, we were unable to adjust for physician’s quality, work hours, education, care level, and specialty. This may lead to an overestimate of physician supply in underserved areas. Physicians in China could be better-educated because of the rising standards for a licensed physician [39], which could be a partial explanation for the lag between the physician supply and population health need and socioeconomic development. Because we did not differentiate between physicians and assistant physicians in our analysis, we were likely to underestimate the disparities in physician density between urban districts and county-level cities, although physician to assistant physician ratios (~ 1.1) are largely consistent across China’s cities [56]. Tertiary care hospitals are more likely to locate in urban districts, and they employ better-educated and more experienced physicians. Consequently, physicians in urban districts are of higher quality than those in county-level cities. Hence, adjusting for the quality of the physician workforce, county-level cities would be of even greater disadvantage in terms of physician density. Furthermore, due to data limitations, we were unable to calculate the number of physicians practicing diverse specialties (e.g., practicing traditional Chinese medicine, pediatricians, and geriatricians) in each city. The health demand for these physicians may not be consistent across cities and regions. Further research is warranted.

Second, physician distribution is only one of the components of health care resources [2]. Other components include the supply of nurses and other health professions, such as primary care providers and specialty physicians, as well as healthcare facilitates. Research on the distribution of a whole range of healthcare resources is warranted. Third, although the present study included several population attributes to reflect population health demand, a fair number of important indicators, such as populations’ comorbid diseases, should be considered.

Forth, our findings based on data from 2003 to 2013 would need to be updated with more recent data. According to the preliminary results from China’s 2020 National Census, the country is aging rapidly, with those aged 60 and over making up 18.70% of the total population, an increase of 5.44% since 2010 [57]. However, urbanization has continued with urban population reaching nearly 64% up from less than 50% in 2010, and China is far more educated with over 200 million populations with university education in 2020, nearly doubled the number in 2010 [57]. Finally, this study did not examine physicians’ availability across China’s rural counties, where a shortage of healthcare resources is even more acute [14]. China differs substantially from other developing and developed nations in terms of city settings, socioeconomic development, and health care systems. To generalize our results, it requires further replications in various contexts under different conditions. Our results contribute to the current body of knowledge on physician distribution and may motivate further studies of the subject.

Despite these limitations, this research represents an improvement over prior research in several respects. First, the distinction between urban districts and county-level cities enabled us to have a deeper understanding of the heterogeneity in physician density in urban China. Second, in measuring physician density, we used the current resident population, including residents and migrants, instead of relying on residents registered in the Hukou system as in more conventional studies. This resulted in a more accurate estimate. Last, our multivariate analyses of longitudinal data are more informative than the descriptive analyses employed in prior studies of physician density in China.

## Conclusion

Despite the reduced inequality in physician distribution between 2003 and 2013, physician supply in China declined after controlling for the growth in population health demand. Urban districts had a higher physician density than county-level cities, but there were regional variations. Future research on physician distribution needs to take into account heterogeneity in population and socioeconomic characteristics.

## Supplementary Information

**Additional file 1: Fig. S1.** Study areas. **Fig. S2.** Physicians per 1000 population in China’s cities (2003, 2013). **Fig. S3.** Lorenz Curves of physician distribution across urban and suburban China in 2003 and 2013. **Table S1.** Population attributes of study areas (mean values), 2003–2013. **Table S2.** Population attributes of urban agglomerations (mean values), 2003–2013. **Table S3.** GEE regression analysis of physician density restricted to cities without administrative promotions from 2003 to 2013, *N* = 611. **Table S4.** Multilevel regression analysis of physician density by grouping urban districts and county-level cities within the provincial/prefectural-level cities, 2003–2013. **Table S5.** GEE regression analysis of physician density excluding four provincial cities, 2003–2013.

## Data Availability

All data came from public domain and could be accessed from CNKI website. 1. China City Statistical Yearbook 2001. 2. China City Statistical Yearbook 2004. 3. China City Statistical Yearbook 2011. 4. China City Statistics Yearbook 2014. 5. National Census Survey 2000. 6. National Census Survey 2010.

## Abbreviations

OR: odds ratio
CI: confidence interval
U.A: urban agglomeration
