# Supplementary material for: Physician distribution across China’s cities: regional variations
Source: Int J Equity Health. 2021 Jul 13;20:162. doi: 10.1186/s12939-021-01503-5 (PMC8276398; doi:10.1186/s12939-021-01503-5)
Supplement: Supplementary file 1 — Additional file 1: Fig. S1. Study areas. Fig. S2. Physicians per 1000 population in China’s cities (2003, 2013). Fig. S3. Lorenz Curves of physician distribution across urban and suburban China in 2003 and 2013. Table S1. Population attributes of study areas (mean values), 2003–2013. Table S2. Population attributes of urban agglomerations (mean values), 2003–2013. Table S3. GEE regression analysis of physician density restricted to cities without administrative promotions from 2003 to 2013, N = 611. Table S4. Multilevel regression analysis of physician density by grouping urban districts and county-level cities within the provincial/prefectural-level cities, 2003–2013. Table S5. GEE regression analysis of physician density excluding four provincial cities, 2003–2013. [file 12939_2021_1503_MOESM1_ESM.doc]

**Supplemental Materials**


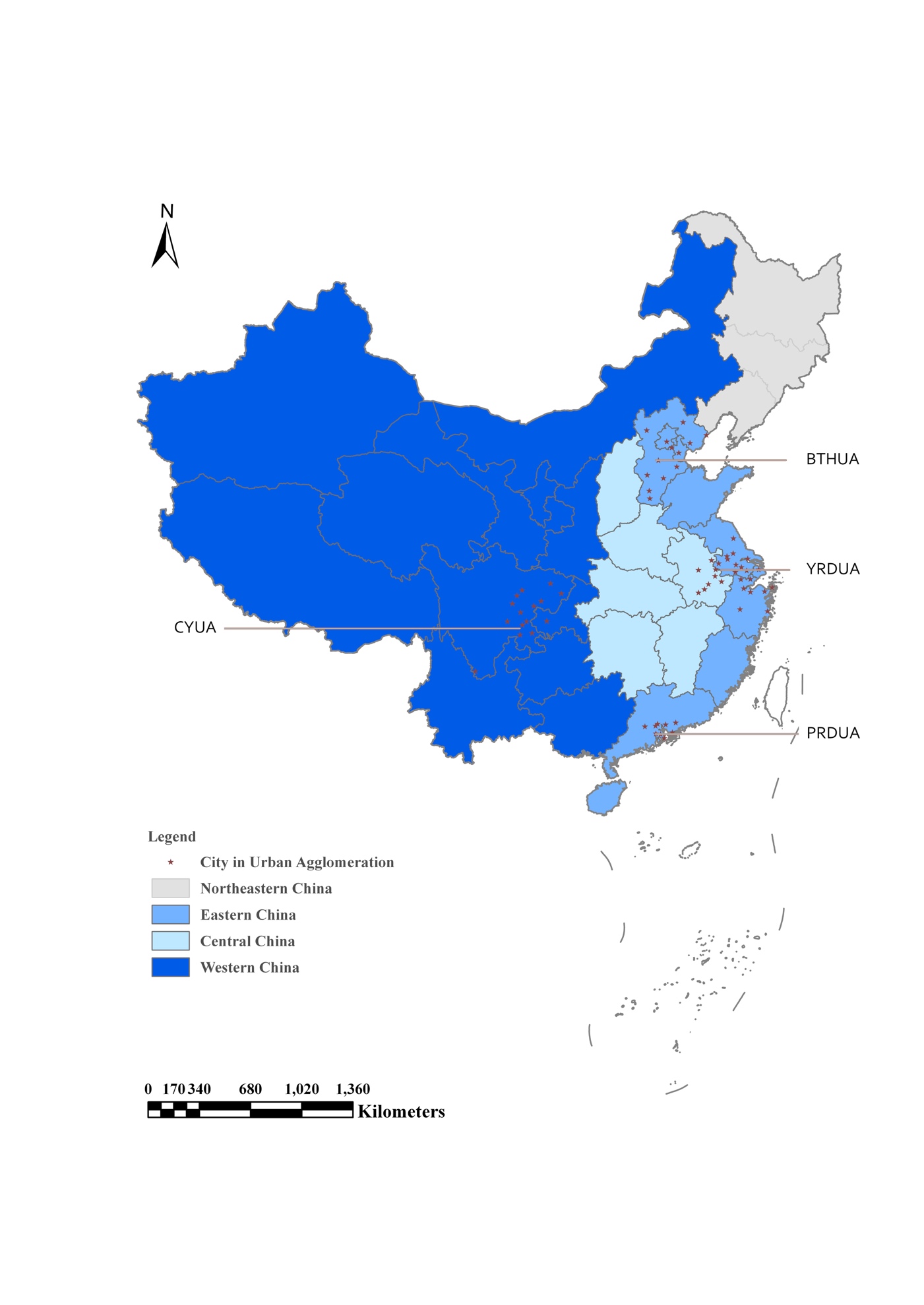


Figure 1 Study areas

Note: BTHUR represents Beijing-Tianjin-Hebei Urban Agglomeration. YRDUR represents Yangtze River Delta Urban Agglomeration. PRDUA represents Pearl River Delta Urban Agglomeration. CYUA represents Chengdu-Chongqing Urban Agglomeration.

**
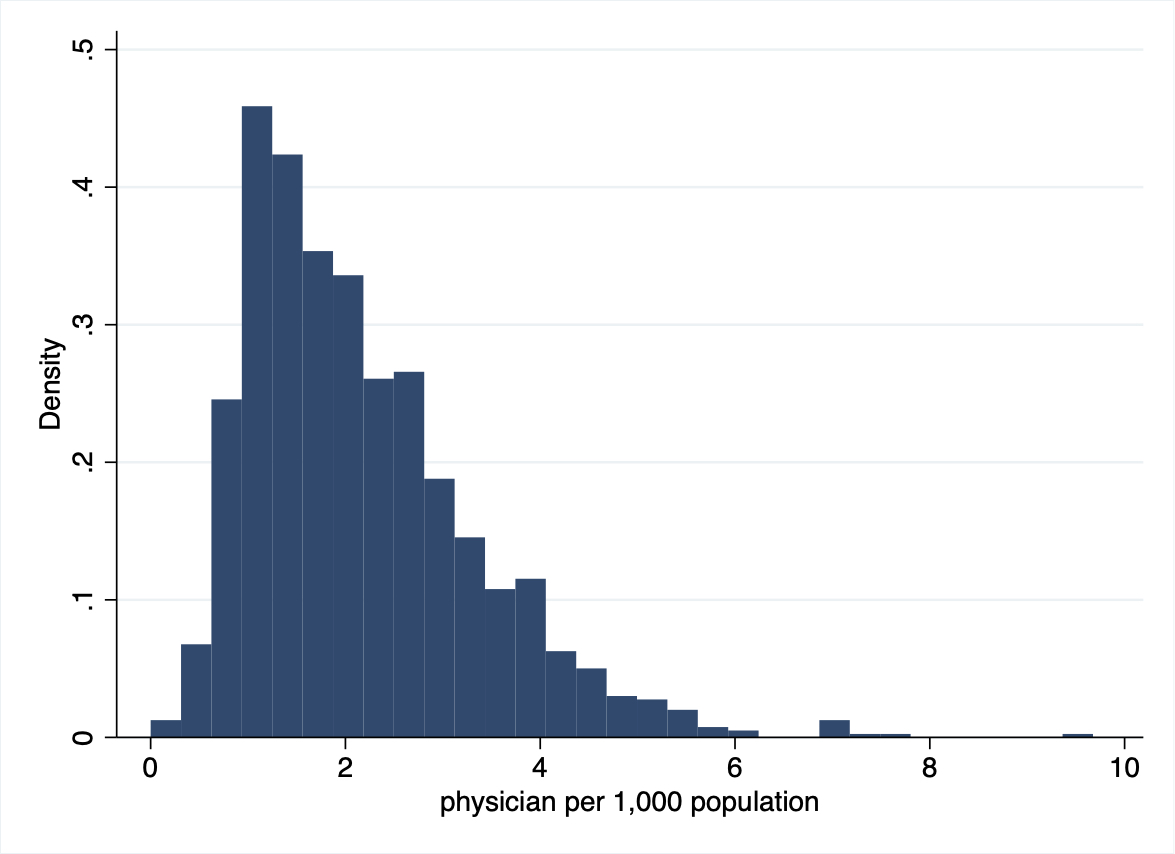
**

Figure 2 Physicians per 1,000 in China’s cities (2003, 2013)


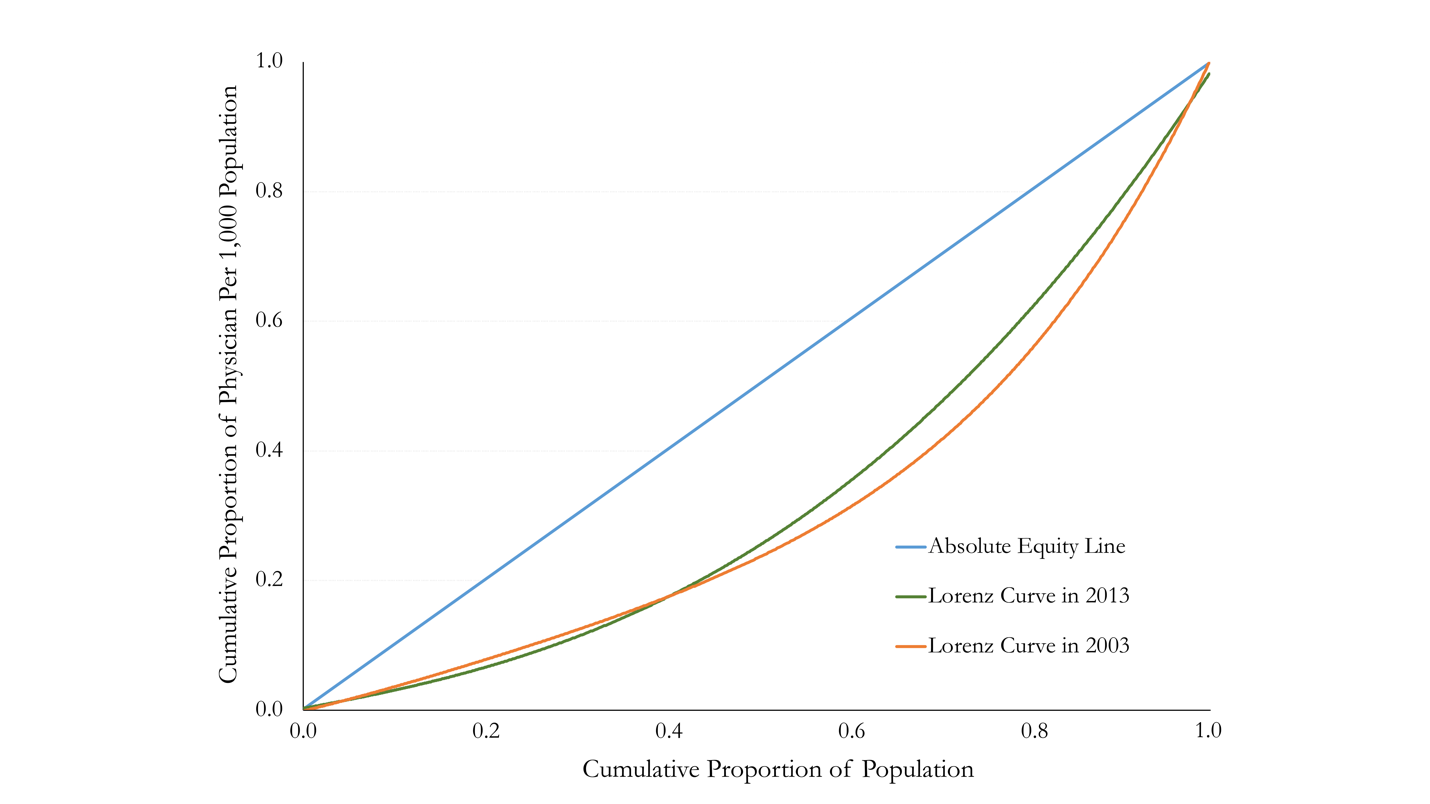


Figure 3 Lorenz Curves of physician distribution across urban and suburban China in 2003 and 2013.

| Table 1. Population attributes of study areas (mean values), 2003-2013 | | | | | |
| --- | --- | --- | --- | --- | --- |
|  |  | 2003 | | 2013 | |
| Region | Variable | Urban district | County-level city | Urban district | County-level city |
| Overall |  | N=272 | N=368 | N=281 | N=357 |
|  | Population density | 1267.92 | 430.10 | 1164.07 | 465.94 |
|  | Female-to-male rate (%) | 94.99 | 94.78 | 95.66 | 101.18 |
|  | Minority (%) | 5.20 | 9.37 | 6.28 | 9.66 |
|  | Aged under 15 (%) | 20.10 | 22.91 | 15.79 | 15.86 |
|  | Aged over 65 (%) | 6.63 | 7.17 | 8.54 | 9.27 |
| Central |  | N=79 | N=88 | N=80 | N=84 |
|  | Population density | 1393.72 | 451.47 | 1309.44 | 455.61 |
|  | Female-to-male rate (%) | 94.53 | 93.35 | 96.64 | 96.07 |
|  | Minority (%) | 2.26 | 2.69 | 2.07 | 2.66 |
|  | Aged under 15 (%) | 20.76 | 24.57 | 15.82 | 17.51 |
|  | Aged over 65 (%) | 6.69 | 6.87 | 8.23 | 8.99 |
| Northeastern |  | N=34 | N=56 | N=34 | N=55 |
|  | Population density | 1010.45 | 175.01 | 845.78 | 173.77 |
|  | Female-to-male rate (%) | 97.51 | 94.99 | 98.75 | 96.54 |
|  | Minority (%) | 6.64 | 15.10 | 6.06 | 13.75 |
|  | Aged under 15 (%) | 16.57 | 19.25 | 10.47 | 12.27 |
|  | Aged over 65 (%) | 6.67 | 6.30 | 9.79 | 9.14 |
| Western |  | N=72 | N=80 | N=84 | N=80 |
|  | Population density | 728.46 | 311.24 | 632.60 | 275.89 |
|  | Female-to-male rate (%) | 92.94 | 92.68 | 95.07 | 94.13 |
|  | Minority (%) | 11.58 | 26.49 | 14.28 | 27.25 |
|  | Aged under 15 (%) | 20.98 | 24.69 | 19.85 | 17.28 |
|  | Aged over 65 (%) | 6.14 | 5.86 | 8.41 | 8.44 |
| Eastern |  | N=87 | N=144 | N=83 | N=138 |
|  | Population density | 1700.76 | 582.26 | 1692.20 | 698.85 |
|  | Female-to-male rate (%) | 96.10 | 96.75 | 94.06 | 110.22 |
|  | Minority (%) | 2.03 | 1.71 | 2.31 | 2.10 |
|  | Aged under 15 (%) | 20.14 | 22.33 | 13.83 | 15.45 |
|  | Aged over 65 (%) | 6.98 | 8.41 | 8.44 | 9.97 |

| Table 2. Population attributes of urban agglomerations (mean values), 2003-2013 | | | |
| --- | --- | --- | --- |
| UA | Variable | 2003 | 2013 |
| Beijing-Tianjin-Hebei U.A. |  | N=11 | N=13 |
|  | Population density (/km2) | 1766.28 | 3542.91 |
|  | Female-to-male ratio (%) | 97.23 | 97.62 |
|  | Minority (%) | 5.10 | 4.41 |
|  | Aged under 15 (%) | 16.98 | 12.42 |
|  | Aged over 65 (%) | 6.72 | 8.21 |
| Yangtze River Delta U.A. |  | N=26 | N=24 |
|  | Population density (/km2) | 2117.73 | 1482.86 |
|  | Female-to-male ratio (%) | 95.29 | 96.10 |
|  | Minority (%) | 0.65 | 1.03 |
|  | Aged under 15 (%) | 16.26 | 11.77 |
|  | Aged over 65 (%) | 8.14 | 9.73 |
| Pearl River Delta U.A. |  | N=8 | N=8 |
|  | Population density (/km2) | 2019.45 | 2098.47 |
|  | Female-to-male ratio (%) | 101.15 | 90.25 |
|  | Minority (%) | 2.08 | 3.00 |
|  | Aged under 15 (%) | 35.09 | 12.30 |
|  | Aged over 65 (%) | 4.13 | 5.09 |
| Chengdu-Chongqing U.A. |  | N=14 | N=15 |
|  | Population density (/km2) | 867.11 | 917.99 |
|  | Female-to-male ratio (%) | 94.53 | 98.97 |
|  | Minority (%) | 0.60 | 0.57 |
|  | Aged under 15 (%) | 19.66 | 14.59 |
|  | Aged over 65 (%) | 8.14 | 10.93 |
| Cities not in any U.A. |  | N=581 | N=578 |
|  | Population density | 689.10 | 659.59 |
|  | Female-to-male ratio (%) | 94.73 | 99.00 |
|  | Minority (%) | 8.20 | 8.82 |
|  | Aged under 15 (%) | 21.91 | 16.15 |
|  | Aged over 65 (%) | 6.90 | 8.93 |
| Note: U.A. represents urban agglomeration | | | |

| Table 3. GEE regression analysis of physician density restricted to cities without administrative promotions from 2003-2013, N=611 | | | | | | |
| --- | --- | --- | --- | --- | --- | --- |
| Characteristics | Model 1  OR (95%CI) | | | Model 2  OR (95% CI) | | |
| Year 2013 (vs. year 2003) | 0.51 | (0.34 to | 0.76)^***^ | 0.50 | (0.33 to | 0.77) ^***^ |
| Urban district (vs. county-level city) | 2.71 | (1.88 to | 3.90) ^***^ |  |  |  |
| Geographic location |  |  |  |  |  |  |
| Eastern (vs. Western) | 0.53 | (0.35 to | 0.79) ^***^ |  |  |  |
| Northeastern (vs. Western) | 0.68 | (0.41 to | 1.13) |  |  |  |
| Central (vs. Western) | 0.64 | (0.43 to | 0.95) ^*^ |  |  |  |
| Geographic location & Administrative level |  |  |  |  |  |  |
| County-level city x Western |  |  |  | ref. |  |  |
| Urban district x Eastern |  |  |  | 1.49 | (0.80 to | 2.76) |
| County-level city x Eastern |  |  |  | 0.33 | (0.20 to | 0.56) ^***^ |
| Urban district x Northeastern |  |  |  | 1.07 | (0.48 to | 2.37) |
| County-level city x Northeastern |  |  |  | 0.58 | (0.30 to | 1.11) |
| Urban district x Central |  |  |  | 1.50 | (0.80 to | 2.80) |
| County-level city x Central |  |  |  | 0.43 | (0.25 to | 0.72) ^**^ |
| Urban district x Western |  |  |  | 1.63 | (0.96 to | 2.75) |
| U.A. (vs. non-U.A.) | 0.88 | (0.50 to | 1.54) | 0.82 | (0.47 to | 1.42) |
| Note: Models 1-2 were adjusted for high school education or above, GDP per capita, population density, female-to-male ratio, migrant, minority, aged under 15, and aged over 65. ^***^ *P* < .001; ^**^ *P* < .01; ^*^ *P* < .05. UA represents urban agglomeration. County-level city x Western represents county-level cities in Western China. GDP was adjusted by Consumer Price Index. | | | | | | |

| Table 4. Multilevel regression analysis of physician density by grouping urban districts and county-level cities within the provincial/prefectural-level cities, 2003-2013 | | | | | | |
| --- | --- | --- | --- | --- | --- | --- |
| Characteristics | Model 1  OR (95%CI) | | | Model 2  OR (95% CI) | | |
| Year 2013 (vs. year 2003) | 0.45 | (0.30 to | 0.68)^***^ | 0.45 | (0.30 to | 0.69) ^***^ |
| Urban district (vs. county-level city) | 2.95 | (2.02 to | 4.30) ^***^ |  |  |  |
| Geographic location |  |  |  |  |  |  |
| Eastern (vs. Western) | 0.47 | (0.28 to | 0.79) ^***^ |  |  |  |
| Northeastern (vs. Western) | 0.76 | (0.41 to | 1.41) |  |  |  |
| Central (vs. Western) | 0.63 | (0.37 to | 1.07) |  |  |  |
| Geographic location & Administrative level |  |  |  |  |  |  |
| County-level city x Western |  |  |  | ref. |  |  |
| Urban district x Eastern |  |  |  | 1.41 | (0.68 to | 2.94) |
| County-level city x Eastern |  |  |  | 0.27 | (0.14 to | 0.53) ^***^ |
| Urban district x Northeastern |  |  |  | 1.11 | (0.44 to | 2.75) |
| County-level city x Northeastern |  |  |  | 0.68 | (0.33 to | 1.41) |
| Urban district x Central |  |  |  | 1.55 | (0.75 to | 3.20) |
| County-level city x Central |  |  |  | 0.40 | (0.20 to | 0.79) ^**^ |
| Urban district x Western |  |  |  | 1.75 | (0.96 to | 3.18) |
| U.A. (vs. non-U.A.) | 1.02 | (0.57 to | 1.81) | 0.86 | (0.47 to | 1.56) |
| Note: Models 1-2 were adjusted for high school education or above, GDP per capita, population density, female-to-male ratio, migrant, minority, aged under 15, and aged over 65. County-level cities and urban districts were clustered within the provincial/prefectural-level cities. ^***^ *P* < .001; ^**^ *P* < .01; ^*^ *P* < .05. UA represents urban agglomeration. County-level city x Western represents county-level cities in Western China. GDP was adjusted by Consumer Price Index. | | | | | | |

| Table 5. GEE regression analysis of physician density excluding four provincial cities, 2003-2013 | | | | | | |
| --- | --- | --- | --- | --- | --- | --- |
| Characteristics | Model 1  OR (95%CI) | | | Model 2  OR (95% CI) | | |
| Year 2013 (vs. year 2003) | 0.49 | (0.33 to | 0.73) ^***^ | 0.50 | (0.33 to | 0.76) ^***^ |
| Urban district (vs. county-level city) | 2.58 | (1.81 to | 3.67) ^***^ |  |  |  |
| Geographic location |  |  |  |  |  |  |
| Eastern (vs. Western) | 0.51 | (0.35 to | 0.75) ^***^ |  |  |  |
| Northeastern (vs. Western) | 0.73 | (0.44 to | 1.19) |  |  |  |
| Central (vs. Western) | 0.63 | (0.43 to | 0.93) ^*^ |  |  |  |
| Geographic location & Administrative level |  |  |  |  |  |  |
| County-level city x Western |  |  |  | ref. |  |  |
| Urban district x Eastern |  |  |  | 1.44 | (0.78 to | 2.65) |
| County-level city x Eastern |  |  |  | 0.32 | (0.20 to | 0.52)^***^ |
| Urban district x Northeastern |  |  |  | 1.06 | (0.48 to | 2.34) |
| County-level city x Northeastern |  |  |  | 0.62 | (0.33 to | 1.16) |
| Urban district x Central |  |  |  | 1.44 | (0.78 to | 2.64) |
| County-level city x Central |  |  |  | 0.42 | (0.25 to | 0.70) ^***^ |
| Urban district x Western |  |  |  | 1.55 | (0.94 to | 2.55) |
| U.A. (vs. non-U.A.) | 0.92 | (0.54 to | 1.59) | 0.79 | (0.46 to | 1.37) |
| Note: Models 1-2 were adjusted for high school education or above, GDP per capita, population density, female-to-male ratio, migrant, minority, aged under 15, and aged over 65. ^***^ *P* < .001; ^**^ *P* < .01; ^*^ *P* < .05. UA represents urban agglomeration. County-level city x Western represents county-level cities in Western China. GDP was adjusted by Consumer Price Index. | | | | | | |
